# A population-based surveillance study on severe acute maternal morbidity (near-miss) and adverse perinatal outcomes in Campinas, Brazil: The Vigimoma Project

**DOI:** 10.1186/1471-2393-11-9

**Published:** 2011-01-22

**Authors:** Eliana Amaral, João Paulo Souza, Fernanda Surita, Adriana G Luz, Maria Helena Sousa, José Guilherme Cecatti, Oona Campbell

**Affiliations:** 1 Department of Obstetrics and Gynecology, University of Campinas (UNICAMP), Campinas, São Paulo, Brazil; 2 London School of Hygiene and Tropical Medicine, University of London, Keppel Street, London WC1E 7HT, UK

## Abstract

**Background:**

Auditing of sentinel health events based on best-practice protocols has been recommended. This study describes a population-based investigation on adverse perinatal events including severe acute maternal morbidity (near-miss), maternal and perinatal mortality, as a health intervention to help improve the surveillance system.

**Methods:**

From October to December 2005, all cases of maternal death (MD), near-miss (NM), fetal deaths (FD), and early neonatal deaths (END), occurring in Campinas, Brazil, were audited by maternal mortality committees.

**Results:**

A total of 4,491 liveborn infants (LB) and 159 adverse perinatal events (35.4/1000 LB) were revised, consisting of 4 MD (89/100.000 LB) and 95 NM (21.1/1000 LB), 23.7 NM for each MD. In addition, 32 FD (7.1/1000 LB) and 28 END (6.2/1000 LB) occurred. The maternal death/near miss rate was 23.7:1. Some delay in care was recognized for 34%, and hypertensive complications comprised 57.8% of the NM events, followed by postpartum hemorrhage.

**Conclusion:**

Auditing near miss cases expanded the understanding of the spectrum from maternal morbidity to mortality and the importance of promoting adhesion to clinical protocols among maternal mortality committee members. Hypertensive disorders and postpartum hemorrhage were identified as priority topics for health providers training, and organization of care.

## Background

Reducing maternal mortality is a target of the Millennium Development Goals. The maternal mortality ratio (MMR) is used to track its trends, and constitutes the most sensitive indicator for social inequalities. It varies from 20 to 920/100,000 live births, resulting in a lifetime risk of death of one per 16 women in Sub-Saharan Africa, contrasting with one per 4,100 in developed countries [1]. Recent evaluation of the progress towards this goal shows that the results so far have been largely insufficient, particularly in the regions where maternal mortality is very high [2].

The Safe Motherhood Initiative, which proposed to stratify care at delivery by classifying cases during pregnancy as being of low or high risk, proved unsuccessful [3]. The initiative that followed, *Making Pregnancy Safer*, was based on the hypothesis that every delivery should be carried out by well-trained professional staff with easy access to emergency obstetrical care [4,5]. Confidential enquiry or audit of cases is a successful strategy to identify problems associated with maternal mortality, adopted by United Kingdom and many other countries [6-8]. In Brazil, the maternal mortality committees, starting in the eighties, were consolidated due to a decree issued by the Ministry of Health, establishing that surveillance of maternal and child mortality was the responsibility of the municipalities and its surrounding regions, as permanent working group requested by the public health system [9]. Nevertheless, between 2000 and 2007, no reduction on maternal mortality ratio was identified, varying from 73.3 to 77/100,000 LB [10].

Known obstacles to reducing the MMR in developing countries include lack of material and human resources, as well as difficulties in accessing services due to financial, geographical and cultural limitations. In the more developed regions, the main obstacles include inadequate use of technology, access to care associated with the organization of the health system and delays in initiating clinical interventions or required procedures [8,11], the last proved to be associated with a significant proportion of cases [12].

Investigation of severe acute maternal morbidity (near-miss) has been proposed in the midst of the discussion on the lack of effectiveness of previous initiatives to reduce maternal mortality [13,14]. Near-miss cases have similar pathways as maternal deaths, with the advantages of offering a larger number of cases for analysis, greater acceptability of individuals and institutions since death did not occur, and the possibility of interviewing the woman herself. Nevertheless, the fact that near-miss cases are much more numerous poses additional challenges, making their investigation more complex [15-17], even if the strategy can be view as rewarding for participant members [18].

Auditing of sentinel health events based on best-practice protocols has been recommended [19]. A review on the effects of auditing and feedback in professional practice and healthcare results concluded that they can be effective, and that their effect is stronger when initial adhesion to good practice is low [20].

Campinas is a city in Sao Paulo State that constitutes a referral center for medical care of a catchment's area comprising 3,971,102 million inhabitants distributed in 42 cities [21]. It locates the headquarters of the municipal and regional committees on maternal mortality responsible for investigating all deaths occurring in region, which are composed by health system and reference maternities managers, and representatives of medical and nursing council [9]. The objective of this study was to describe a population-based investigation of adverse perinatal events, including severe acute maternal morbidity (near-miss), maternal and perinatal mortality as a health intervention to help improve the surveillance system.

## Methods

This was a descriptive, population-based study, that investigated all cases of severe acute maternal morbidity or near-miss (NM), maternal deaths (MD) and perinatal deaths (including fetal death - FD and early neonatal death - END), occurring in the city of Campinas between October and December, 2005. Inclusion criteria consisted of maternal deaths and near-miss cases occurring up to 42 days after delivery, fetal death at 500 grams or more (or ≥ 22 weeks of pregnancy), and early neonatal deaths, before the newborn infant had completed 7 full days of life [22].

It was proposed as a health system intervention collaborative project between academia and the municipal and regional maternal mortality committees named Vigimoma (*Vigilância de Morbidade Materna/*Maternal Morbidity Surveillance), contributing to build capacity among both municipal and regional committees' members on revising all perinatal adverse events together.

Near miss cases were defined as cases in which acute organic dysfunction occurred that, if not appropriately treated, would have resulted in maternal death. Specific near-miss criteria were adapted from Mantel et al. [13] and Waterstones et al. [23]: Admission to an intensive care unit (ICU); Transfer due to severe clinical condition; Coma; Shock (systolic pressure < 80 mmHg); Cardiorespiratory arrest; Hysterectomy/laparotomy; Blood transfusion ≥ 3 units of red blood cells, plasma or whole blood; Respiratory support other than during anesthesia; Need for O_2 _therapy other than during anesthesia; Need for procedures of peritoneal or intravenous dialysis; Oliguria for more than 12 hours; Clinical uraemia or urea > 100 mg/dl; Hypertensive crisis (diastolic pressure ≥ 110 mmHg with symptoms); Congestive heart failure; Acute pulmonary edema; Use of fluids (> 2 L) or medication to expand blood volume; Eclampsia and/or HELLP syndrome; Systemic inflammatory response syndrome (SIRS); and Adult Respiratory Distress Syndrome (ARDS). This study was conducted before the World Health Organization has defined maternal near miss and their diagnostic criteria, but most of these criteria are also included in the current WHO definition of maternal near miss [24].

In Brazil, total fertility rate declined to 1.8 children per women from 2002-2006 [25]. There are 97.46% hospital-based deliveries, reaching 99.46 in Sao Paulo State, where Campinas is located, with 99.7% deliveries occurring in public or private hospitals [26]. For this study, data on adverse perinatal events from nine maternity services (corresponding to 98% deliveries) were included: three hospitals concentrate the majority of deliveries and also high-risk cases (one exclusively university-public, and two others attending public and insured/private patients) and the remaining six attend lower risk and minority of deliveries (insured or private patients).

A team consisting of an obstetrical nurse and a doctor from these three largest maternity hospitals visited the wards, intensive and emergency units daily, collecting data from the medical and administrative records, with no interview. After three month, the same trained teams retrospectively reviewed clinical and administrative data from other maternity hospitals included.

Clinical care provided in each case was compared with the standards defined in the Manuals on Emergencies and Humanized Healthcare during Delivery, Abortion and in the Puerperium, issued by the Ministry of Health [26,27]. These activities were supervised by two research assistants and the principal investigator. After the data had been collected, the cases were discussed anonymously by the Municipal Committee on Maternal Mortality in the case of women living in Campinas, and by the Regional Health Directorate Committee in the case of women residing within the catchments area. Committees' members identified potential delays in seeking, reaching or receiving care (presented as percentages). A preventability score (PS) was proposed, based on the potential capacity of each intervention suggested to prevent the situation, ranging from 0 (none) to 5 (max) as judged by committee members in a participatory, consensus-driven process. The mean PS was calculated from PS for each individual intervention, and the total PS per event was proposed as a product of the mean score of each intervention multiplied by the number of cases for which it was suggested.

The maternal deaths occurring during the study data collection period were cross checked using the official vital registries. The number of deaths in the study period was confirmed from the official records. Ratios per 1000 live birth were calculated using the information obtained from the national Live Birth Information System (SINASC).

The research committee of CAISM/UNICAMP, the institutional review board of the School of Medical Sciences, the Regional Health Directorate, the Municipal Health Department, and the institutional review boards of the hospitals participating in the auditing process approved the research protocol. Anonymity of all cases (women and neonates), of the institutes and of the individual staff members involved was maintained.

## Results

In total, there were 13 four-hour meetings with 23 members for the Municipal Committee, and 7 four-hour meetings with 20 similar members for the Regional Committee, with an average of 30 minutes per case for discussion.

One hundred and fifty-nine adverse perinatal events occurred in the 4,491 live-births (35.4/1000 LB) registered for the studied trimester. Cases of NM corresponded to 60.1% of all events, or 21.2 NM/1000 LB, while maternal death comprised 2.5% of cases, with 23.7 NM for each MD (Table 1).

**Table 1:** Cases of adverse perinatal events in Campinas, October to December, 2005

Perinatal events	Observed(n)	%	per 1000 LB	Expected during project phase (n)
Maternal death	4	2.5	0.89	3
Near-miss	95	59.7	21.2	47
Perinatal death	60	37.7	13.4	72
Fetal death	32	20.1	7.1	36
Early neonatal death	28	17.6	6.2	36
Total	159	100.0	35.4	122

Among all cases, slightly more than half (58.4%) lived in Campinas, 14% were adolescents, and one-fifth had more than 35 years of age. Almost half the women had no professional activity. For a proportion, the education level was not registered, but the majority of the remained had attended school up to the 8^th ^grade. Three-quarters of the women lived with a steady partner, and had low parity. Most have done regular antenatal visits, but only 16.6% had private care (Table 2).

**Table 2:** Characteristics of women with adverse perinatal events in Campinas, October to December, 2005

Characteristics	N	%
Live in Campinas	93	58.5
Age < 20 years	22	13.8
Studied up to 4^th ^grade	16	10.1
Live with steady partner	118	74.2
No professional activity outside the home	68	42.8
Current pregnancy first or second	104	65.4
Received prenatal care	136	85.5
Private or insured antenatal care	26	16.4

Complications occurred during pregnancy in 47.7% of these women, mainly hypertension (40 cases), valve or myocardial diseases (21 cases), urinary tract infection (11 cases) and diabetes (7 cases). The diagnosis of preeclampsia or hypertension was registered for 48% of cases, but prior admission to hospital for only 17.9%. Almost three-fourth adverse perinatal events were identified during labor, or post partum.

The four deaths occurred due, respectively, to central nervous system bleeding complicating anticoagulation therapy, hemorrhage due to placenta previa, and also septicemia following fatty liver disease, and another complicating urinary tract infection. In the cases of near-miss, hypertensive disorders predominate, followed by postpartum hemorrhage and heart failure.

Among the cases of fetal death, intrauterine anoxia was the most frequent immediate cause, followed by malformations. Among the early neonatal deaths, respiratory failure due to prematurity was the most common cause. Maternal hypertensive complications were present in the case of one-third of stillborn infants and in one-sixth of cases of neonatal death (Table 3).

**Table 3:** Main causes of adverse perinatal events in Campinas and the percentage of the cause within the group of the event

Causes	n	% of the event within the group
*Maternal death*		
Central nervous system bleeding complicating anticoagulation therapy	1	25
Hemorrhage due to placenta previa	1	25
Septicemia following fatty liver disease	1	25
Complicating urinary tract infection	1	25
*Near-miss*		
Hypertensive complications	54	57.8
Postpartum hemorrhage	17	17.9
Congestive heart failure	6	6.3
Sepsis	3	3.1
Others	15	15.8
*Fetal death*		
Intrauterine anoxia	15	46.9
Malformations	8	25.0
No information	9	28.1
*Early neonatal death*		
Respiratory failure	13	46.4
Sepsis	4	14.3
Anoxia	3	10.7
Malformation	3	10.7
Others	5	17.8

Near misses, maternal death and perinatal death occurred predominantly in the exclusively public university maternity service, which attended all four maternal deaths, 48% of NM cases, 41% of fetal deaths and 43% of neonatal deaths. Less than one-quarter of cases contained registered information on being transferred from other institution. Among those informed, only one-quarter had been referred by the previous attending doctor.

The intervention measures proposed and a score on potential impact for the prevention of similar cases were summarized in Tables 4 and 5. For maternal death, better management of obstetrical hemorrhage could improve care. For maternal morbidity, the same intervention toped as the mean score attributed, but improving the use on magnesium sulphate for preeclampsia reached the higher total score due to the frequency of the clinical condition and intervention suggested by the committees' members.

**Table 4:** Principal interventions proposed for the prevention of maternal death and near-miss, and potential preventability score (0-5) assigned by the maternal death committees

	Frequency (n)	Mean score (± SD)	Total Score*
**To avoid maternal death**			
Provide training in managing obstetrical hemorrhage	2	5.0 (± 0.0)	10
Educate pregnant women about warning signs during pregnancy	2	4.0 (± 1.4)	8
Provide access to blood products in secondary hospitals	1	4.0 (± 0.0)	4
Perform ultrasound in the 3 rd trimester if risk of placenta previa	1	4.0 (± 0.0)	4
Improve care with adhesion to protocols into hospital	2	3.5 (± 0.7)	7
Provide safe transportation to the referral hospital	1	3.0 (± 0.0)	3
Improve the diagnosis and management of urinary infections	1	2.0 (± 0.0)	2
**To avoid maternal near-miss**			
Promote family planning if severe clinical disease	5	5.0 (± 0.0)	25
Promote prophylaxis for postpartum hemorrhage	6	4.8 (± 0.4)	29
Train staff to use magnesium sulphate in preeclampsia	15	4.2 (± 0.6)	63
Train in the management of preeclampsia and arterial hypertension	11	4.0 (± 0.6)	44
Promote the diagnosis of cardiac diseases during pregnancy	5	3.8 (± 1.1)	19
Improve clinical practice in prenatal care with adhesion to protocols	12	3.7 (± 0.8)	44
Educate pregnant women about prenatal warning signs	7	3.4 (± 1.4)	24
Promote family planning in general	9	3.2 (± 1.7)	29
Train in the management of obstetrical hemorrhage	7	3.1 (± 1.2)	22
Promote use of corticosteroids for fetal pulmonary maturity	5	2.8 (± 1.1)	14
Promote the importance of prenatal care and the need to initiate it early	10	2.6 (± 1.1)	26
Promote postpartum discharge under stable clinical conditions	5	2.6 (± 2.2)	13
Qualify information on medical records	7	2.3 (± 1.0)	16
Implement the flow of counter-reference to the units of origin	6	2.0 (± 0.9)	12
Add information on prenatal care to the patient's medical records	6	2.0 (± 1.6)	12
Promote postpartum reproductive counseling	6	1.7 (± 0.6)	10

*Total score: mean score attributed x number of cases

**Table 5:** Main interventions proposed for the prevention of fetal death and early neonatal death, and potential preventability score (PPS) assigned by the maternal death committees

	Frequency (n)	Mean Score (± SD)	Total Score
**To avoid fetal death**			
Educate pregnant women about prenatal warning signs	2	4.0 (± 1.4)	8
Improve prenatal clinical practice (adhesion to protocols)	3	3.7 (± 1.1)	11
Promote the use of folic acid if previous neural tube closure defect	2	3.5 (± 0.7)	7
Qualify anatomopathological report in perinatology	3	3.3 (± 0.6)	10
Promote investigation into neonatal and intrauterine death	2	3.0 (± 2.8)	6
Promote postpartum counseling with genetic evaluation	3	2.0 (± 2.6)	6
Promote the importance of prenatal care and its early initiation	5	1.8 (± 1.3)	9
Promote postpartum counseling	4	1.5 (± 1.0)	6
Register information on prenatal care to the patient's medical records	2	1.5 (± 0.7)	3
Promote family planning	3	1.0 (± 1.0)	3
**To avoid early neonatal death**			
Prepare a team for neonatal heart surgery	2	5.0 (± 0.0)	10
Promote the prophylaxis of neonatal disease by group B streptococcus	3	4.0 (± 1.0)	12
Promote protocol for the induction of fetal lung maturity	3	3.5 (± 0.7)	11
Improve prenatal clinical care (adhesion to protocols)	6	2.8 (± 1.5)	17
Improve clinical care for newborn infants (protocols)	2	2.5 (± 0.7)	5
Promote intrauterine diagnosis of malformations	2	2.5 (± 3.5)	5
Promote postpartum counseling	4	2.2 (± 1.3)	9
Promote the importance of prenatal care and its early initiation	7	2.0 (± 1.4)	14
Prevent adolescent pregnancies	2	2.0 (± 0.0)	4
Qualify medical records	2	1.0 (± 0.0)	2

SD: Standard deviation/Total score: sum of the scores attributed to the mean

Some delay was identified among 34.2% of cases, as identified by Committees' members revising the clinical and administrative notes. For 20%, there was a delay in receiving care and for 14.5% seeking care took longer than expected, while reaching care delay was identified for 4.4% - Table 6.

**Table 6:** Delays in care for adverse perinatal events

Delays	N	%
None	93	58.9
Yes (not mutually exclusive)	54	34.2
*Delay in seeking care*	23	-
*Delay in reaching the institute*	7	-
*Delay in receiving care*	32	-
Unknown	12	6.9

## Discussion

This study showed that cases of maternal near-miss are 24 times more frequent than maternal death, occurring at a ratio of 21.1/1000LB, coinciding with the number from the 2006 National Household Survey, using five criteria to identify maternal morbidity in Brazil [28]. This intervention project also showed that the analysis of unsuccessful perinatal events (near miss, maternal death, and perinatal death) is possible when local and regional surveillance committees' routine is integrated with research initiatives.

Studying these cases was an opportunity to review the wide spectrum of clinical conditions that threaten the life of a pregnant woman, and provided additional data for the members of the municipal and regional committees. Their participation was essential to build capacity on clinical thinking causality and preventability of morbidity and mortality of these unsuccessful perinatal events, with the perspective of health managers at system and hospital level, as reported previously [18].

The importance of the quality of clinical care was emphasized, including adhesion to clinical protocols, particularly when dealing with hypertensive complications. The widest scope of interventions arose from the analysis of near-miss cases and the total score reflected the potential greater impact of including the revision of near miss cases.

The prevalence of NM identified was greater than expected when criteria only based on organ function are applied, but it is in agreement with the World Health Organization (WHO)'s systematic review (0.01-2.99%) using criteria of case management, and with other systematic reviews [29-31], but lower than in Bolivia (5% or 50.1/10,000) where clinical and management criteria were applied [32].

The implementation of any intervention and its reevaluation through another process of data collection closes the so-called auditing cycle. There is evidence from systematic reviews of the effectiveness of auditing interventions, particularly when current adhesion to the recommended practices is inadequate [20]. The need to standardize management and to follow protocols was the most frequent recommendation in the discussions of cases by the committees.

The finding that hypertension in pregnancy was the main complication responsible for the adverse perinatal events is in agreement with the WHO systematic review on maternal death in Latin America and a study from Bolivia, but differs from Africa and Asia where hemorrhage is more prevalent. Hypertension was also the single most common cause in developed countries (16.1%), behind all the direct causes together, and ahead of embolia and hemorrhages [31,32]. This profile shows that the pattern of perinatal morbidity in the Campinas region is similar to developed countries; therefore, strategies and interventions need to be adapted. Adding surveillance of near-miss cases to qualify the system of confidential enquiry and auditing is an important step in this direction.

The finding that 48% of patients were referred from other municipalities as well as from the supplementary health system reflects the need to organize perinatal care regionally, involving public and private healthcare managers, maternity hospitals and staff. To reach this widely encompassing involvement is an enormous challenge. Some professional institutions, and various instances of the National Health System are important catalyzing elements in the process and their involvement must be assured. Among the important elements there are management commissions between hospitals and the Municipal Department of Health, maternity clinical directors, the bipartite committee of the Regional Health Directorate (municipal and state representatives), the State Association of Specialists in Gynecology and Obstetrics, and the Regional Medical Council. This need for a more participative involvement to reduce maternal mortality, particularly from the professional associations, has been emphasized previously by other authors [33].

One of the difficulties in investigating maternal deaths in Brazil is gaining visibility for the results of the work and, consequently, credibility for the intervention proposals, making their implementation feasible first hand. The current surveillance system has been unable to mobilize health system managers and healthcare professionals working in perinatology as expected. The experience gained in carrying out this study served as training for the members of the committees in the auditing process and motivated them towards carrying out a more purposeful investigation that included many hours of discussion in addition to their regular activities, as observed in other places [18].

Nevertheless, maintaining a near-miss surveillance system must not be dependent on the motivation of this group of technical staff [34]. It has to depend on the political commitment of the managers and staff of the institutions and on the health system to provide support for revising the events, implementing and evaluating the healing interventions, thereby ensuring full auditing process within the routine clinical activities. The close collaboration between the university and the health services in carrying out this project was essential to ensure its acceptance in the maternity hospitals. The challenge to maintain the involvement of these entities include ample participation on the interventions proposed, many of which including workshops on evidence-based clinical protocols.

There is no widely spread tradition of involving professional medical associations in these qualification processes except through scientific events. This multi-institutional involvement is one of the factors associated with the success of any maternal healthcare qualification proposal [35]. In the United Kingdom, the participation of obstetricians in auditing procedures is a prerequisite of the professional association itself [36]. Collaboration between universities and services may work towards motivating the necessary change.

Interventions that qualify healthcare during delivery and childbirth have been found to be effective, as confirmed by auditing procedures with long-term reevaluation. Mercer et al. [37] in Northern India, trained teams to use oxytocin and ergometrine to prevent hemorrhage in the third stage of labor, and to introduce partographs to accelerate the decision-making process to transfer cases for Caesarean section during intrapartum care, which in the 7^th ^year of audit had reduced postpartum hemorrhages by 50% and led to more rational decision-making with respect to transfer during labor.

As seen from our data, no delay was incurred in almost 60% of cases. In the remaining cases, the most common finding was a delay in receiving adequate care, often because a particular protocol had not been adopted by that institution. From 2003-5 in United Kingdom, delayed care was recognized as a relevant factor among 17% of women, but substandard care could be pointed in 64% of maternal deaths due to direct causes and 40% due to indirect causes [36]. Similar rates were found for severe maternal morbidity in the Netherlands [38]. Since there is no further information on the criteria used for classification, the rates published may well include less than adequate care, which may not be directly responsible for the near miss or death, as highlighted by Scottish classification [36]. Anyway, this confirms the need to discuss and implement good clinical practice protocols as the most effective measure with the greatest potential impact. Management of hypertensive complications and puerperal hemorrhages emerged as the top clinical conditions priorities for the region.

In this study, the members of the committees had to review the suggestions for improvement in each individual case and the potential impact of these suggestions in reducing the occurrence of a similar adverse event. The exercise of thinking about the causal pathway for the unsuccessful event as a separate exercise from identifying the aspects of substandard care motivated the learning process of the group. This process also contributed towards emphasizing the importance of the knowledge and the publication of the recommendations based on scientifically-based protocols, such as the Manuals for Care during Delivery, Abortion and the Puerperium [25] and the Ministry of Health's Obstetrical Urgencies and Emergencies Manual [26], used as reference for this project. The need to incorporate scientific evidence into the audit procedures and to use clinical protocols and training programs to improve the quality of obstetrical care, as experience by the group, has been mentioned before by other authors [39,40].

This study involved the intensive surveillance of cases in the institutions over a short period of time, and was made possible with supplementary financial support for the data collection teams, and unpaid extra-hour contribution of the members of the committees. Without abandoning the confidential enquiry into all maternal deaths, we suggest the evaluation of near-miss as a sentinel event in perinatal health in a cross-sectional study design, for a short, intensive period of 1-3 months, putting municipal and regional committees together to discuss interventions expected to improve perinatal care. The impact of this program has to undergo reevaluation with the support and articulation of the participating institutions, including the university, and professional organizations.

## Conclusions

Despite the large number of cases, the investigation into near-miss events is feasible and was the source of motivation for the participants of the committees on maternal mortality, giving visibility to the wide spectrum of causes of maternal morbidity. Hypertensive complications and postpartum hemorrhage were identified as priority subjects for training in prenatal care and healthcare during childbirth, respectively. This study confirms the importance of adhesion to best-evidence-based clinical protocols. The challenge remains to involve professionals working in the institutions in the processes of auditing, and to ensure the participation of the professional associations. This experience and other studies public after showed that systematic and frequent revision of these cases could be incorporate into the continuum of the health surveillance system.

## Competing interests

The authors declare that they have no competing interests.

## Authors' contributions

EA was responsible for conceiving and implementing the project, data collection and analysis, facilitation of committees' meetings, writing and revising the manuscript; JPS contributed on implementation, data collection at hospital level and committee meetings, preparation and analysis of the data, as well as drafting and revising the manuscript; FS and AGL facilitated committees' meetings and data collection, and revised the manuscript; MHS helped on project design, data analysis, drafting and revising the manuscript; JGC and OC participated on project design, analyzing the data, drafting and revising the manuscript. All authors approved the final version of the manuscript.

## Pre-publication history

The pre-publication history for this paper can be accessed here:

http://www.biomedcentral.com/1471-2393/11/9/prepub
